# Excess cases of prostate cancer and estimated overdiagnosis associated with PSA testing in East Anglia

**DOI:** 10.1038/sj.bjc.6603246

**Published:** 2006-07-11

**Authors:** N Pashayan, J Powles, C Brown, S W Duffy

**Affiliations:** 1 Department of Public Health and Primary Care, Institute of Public Health, University Forvie Site, Robinson Way, Cambridge CB2 2SR, UK; 2 Eastern Cancer Registration and Information Centre, Addenbrookes, Cambridge CB2 2QQ, UK; 3 Cancer Research UK, Centre for Epidemiology, Mathematics & Statistics, Wolfson Institute of Preventive Medicine, London EC1M 6BQ, UK

**Keywords:** excess cases, overdiagnosis, PSA, prostate cancer

## Abstract

This study aimed to estimate the extent of ‘overdiagnosis’ of prostate cancer attributable to prostate-specific antigen (PSA) testing in the Cambridge area between 1996 and 2002. Overdiagnosis was defined conceptually as detection of prostate cancer through PSA testing that otherwise would not have been diagnosed within the patient's lifetime. Records of PSA tests in Addenbrookes Hospital were linked to prostate cancer registrations by NHS number. Differences in prostate cancer registration rates between those receiving and not receiving prediagnosis PSA tests were calculated. The proportion of men aged 40 years or over with a prediagnosis PSA test increased from 1.4 to 5.2% from 1996 to 2002. The rate of diagnosis of prostate cancer was 45% higher (rate ratios (RR)=1.45, 95% confidence intervals (CI) 1.02–2.07) in men with a history of prediagnosis PSA testing. Assuming average lead times of 5 to 10 years, 40–64% of the PSA-detected cases were estimated to be overdiagnosed. In East Anglia, from 1996 to 2000, a 1.6% excess of cases was associated with PSA testing (around a quarter of the 5.3% excess incidence cases observed in East Anglia from 1996 to 2000). Further quantification of the overdiagnosis will result from continued surveillance and from linkage of incidence to testing in other hospitals.

A descriptive epidemiological study in East Anglia has shown a 6% excess of prostate cancer registrations during 1991–2000 relative to expectations based on pre-1991 trends, coincident with an increase in prostate-specific antigen (PSA) testing (Pashayan *et al*, 2006). This increase in PSA testing came not from a formal screening programme (no such programme existed), but rather from the increased use of the test for case finding and in the investigation of men with urological symptoms. It is of interest to estimate how many of the excess cases are attributable to PSA testing and how many of these are plausibly classed as overdiagnosed.

Overdiagnosis is usually defined conceptually as the diagnosis, as a result of screening, of a cancer usually histologically confirmed, which would have not achieved clinical significance during the lifetime of the host had screening not taken place (Paci *et al*, 2004). Here, potential overdiagnosis would be attributed to increased diagnostic testing rather than to screening. The prevalence of urological symptoms in males increases to high levels in late middle and old age. In recent years, the advent of PSA testing has led to a fast and relatively simple diagnostic sequence for prostate cancer. This may have occasioned diagnosis of the disease in some elderly men who would not have had such a diagnosis in their remaining lifetime in the absence of PSA testing. Thus, a phenomenon analogous to screening-induced overdiagnosis may take place as a result of diagnostic testing.

In this study, we examine the relationship between PSA testing and prostate cancer diagnosis in a residentially defined population from 1996 to 2002, and estimate the extent to which overdiagnosis has contributed to the 6% excess in East Anglia.

## MATERIALS AND METHODS

### Estimation of the population of the Cambridge area

The population of interest was defined by residence using postcode districts (first three or four characters of the 1998 Postcode address). Men living in postcode areas CB1 to CB4, which are completely within the administrative boundaries of Cambridge City (CC) and South Cambridgeshire (SC), were included. Men living within the defined postcodes constituted 92% of CC and 72% of SC total male district populations. This area is hereafter referred to as the Cambridge area. Assuming homogeneity between the included and excluded populations, the age profile of the included population could be estimated. This was the only population for which individual testing information was available.

Individual PSA testing records from 1996 were available at Addenbrookes Hospital, which serves the Cambridge area. Using the NHS number, the laboratory linked its records of PSA tests to the Eastern Cancer Registry and Information Centre's (ECRIC) records of prostate cancer diagnoses in residents of the Cambridge area between 1996 and 2002. The linked records made it possible to identify whether a test was performed before diagnosis, in the peridiagnostic period (see below) or for monitoring prostate cancer postdiagnosis. The PSA tests with no match among prostate cancer registrations were considered not to have led to a cancer diagnosis.

### Statistical analysis

Prediagnosis PSA testing was defined as PSA testing at least 6 months before any diagnosis of prostate cancer. Peridiagnostic testing was defined as any PSA testing before the date of pathological diagnosis, including within the 6 months before diagnosis. Thus, this would include PSA tests that led directly to the diagnosis.

In the primary analysis, a Poisson regression model was fitted with cases of prostate cancer and prediagnosis PSA testing status, adjusting for the effects of age and calendar year. Deviance *χ*^2^ statistics were used to determine the significance of the effects of the variables (prediagnosis PSA testing, age, year) and to examine the interaction between age and PSA testing, that is, to see whether the association between PSA testing and prostate cancer diagnosis differed by age.

In sensitivity analysis, the definition of prediagnosis was relaxed to include peridiagnosis PSA testing.

For purpose of this study, we define overdiagnosis as the diagnosis of asymptomatic prostate cancer through PSA testing, whose lead time is such that the host would have died before serious clinical problems arose. If the mean sojourn time (lead time) is *M*, the probability that a PSA-detected case would have taken longer than the remaining lifetime to become symptomatic can be estimated as (−*t*/*M*)where *t* is the expected remaining lifetime (Duffy *et al*, 2001; Paci *et al*, 2004).

Using expected remaining lifetimes for the UK male population in 2001–2003, and estimates of lead time from the literature, age-specific probabilities of overdiagnosis were calculated. Applying these probabilities to the number of cases with prediagnosis PSA test, the number of overdiagnosed cases and therefore the proportion of the recent excess cases considered to be overdiagnosed were estimated. Excess cases refer to prostate cancer patients whose diagnosis at the time was likely to be attributable to PSA testing activity.

Analysis was performed using STATA version 7.0. *P*<0.05 was considered statistically significant.

## RESULTS

### PSA tests in the Cambridge area

In the Cambridge area from 1 January 1996 to 31 December 2002 (that is the period during which we had PSA data linked to cancer registry data), 8894 men had at least one PSA test, of whom 23% (2053) were 50–59 years, 30% (2701) were 60–69 years and 38% (3352) were 70–89 years. Sixty-four percent (5722) of men had repeat peridiagnosis PSA testing. Only 38 of these men with peridiagnosis PSA testing had a prostate cancer diagnosis. The crude proportion of men at risk – 40 years or over, with no known prior diagnosis of prostate cancer – having a PSA test in a given year increased from 1.4% in 1996 to 5.2% in 2002.

**Figure 1:**
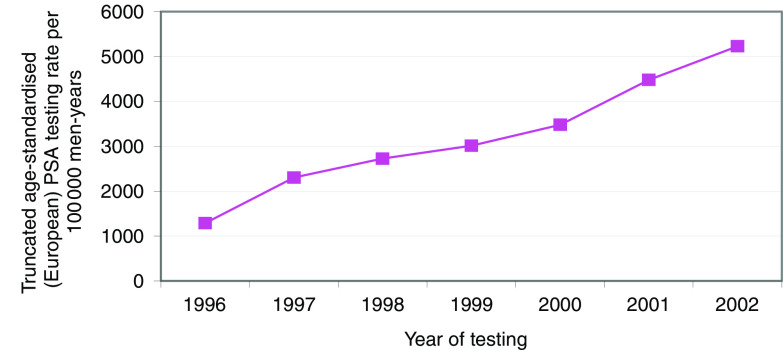
Prostate-specific antigen testing rate per 100 000 men-years 40 years or over with no previous cancer diagnosis, Cambridge area, 1996–2002, standardised using the truncated European Standard Population.

Figure 1 shows the age-standardised testing rate in the Cambridge area residents from 1996 to 2002.

### Excess cases associated with PSA testing

Table 1 gives the number of cases, person-years and incidence rates of prostate cancer by previous testing status and age. As expected, increased incidence was observed at older ages. One can also see the PSA testing rate increasing with age in this table. The men-years in the tested category rose from 0.6% at age 40–44 years to 72% at age 85–89 years. The crude incidence of registered prostate cancer was 360 out of 100 000 men-years in men who had a PSA test before diagnosis, and 248 out of 100 000 men-years in men with no prediagnosis PSA test. The increase in reported incidence was most evident in the age range 50–64 years, with higher incidence among those not tested at older ages.

The rate ratios (RR) from Poisson regression associated with PSA testing history are shown in Table 2, both unadjusted and adjusted for calendar year and age. Unadjusted, there was a significant 45% increase in risk associated with PSA testing. The effect of PSA testing was no longer observed when adjusted for age (age-adjusted RR=0.86, 95% confidence intervals (CI) 0.60–1.23). There was heterogeneity by age of the effect of PSA testing (*P*<0.001). Analysis separately for age strata 40–64 and 65–89 years showed that PSA testing was associated with more than three-fold increase in risk (*P*=0.001) in the younger groups, and there was a nonsignificant reduction in risk associated with PSA testing in the older groups. Thus, neither the unadjusted nor the age-adjusted effect of PSA testing is ideal. For simplicity we use the unadjusted effect below.

### Estimation of overdiagnosis

Table 3 shows lead time estimates from the literature for screen detected prostate cancer. The majority of the estimates lie in the range 5–10 years. We assumed that these would generalise to cancers detected by PSA testing outside formal screening programmes. We therefore performed three analyses of lead time, assuming average sojourn time (duration of the preclinical screen detectable period) of 5, 7 and 10 years. The age-specific probabilities of overdiagnosis corresponding to each lead time and the estimated numbers of overdiagnosed cases are shown in Tables 4 and 5, respectively.

The number of excess cases among the PSA-tested men was calculated using the unadjusted RR of 1.45, estimated from the Poisson regression. For example, for the age group 80–84 years, the figure of three cases is calculated to be 45% higher than the incidence would have been in the absence of PSA testing. The excess is calculated as 3−(3/1.45)=0.93.

It was estimated that 10 (31%) out of 32 cases were excess cases. Depending on the assumed lead time of 5, 7 and 10 years, 40, 60 and almost 100%, respectively, of the excess cases were estimated to be overdiagnosed cases (Table 5).

In a sensitivity analysis, the definition of prediagnosis was relaxed to peridiagnosis, including any PSA test performed up to the date of diagnosis. This analysis gave 38 cases with peridiagnosis PSA. The unadjusted RR of cancer diagnosis in men with PSA test record *vs* men with no PSA test record was 1.72 (95% CI 1.24–2.39). The interaction with age was still observed. The age-specific RR for men younger than 65 years was 4.47 (95% CI 2.41–8.31) and 0.77 (95% CI 0.53–1.14) for men 65 years or over. The estimated number of overdiagnosed cases in the Cambridge area for a 7-year lead time increased from 6 to 8.

## DISCUSSION

The proportion of men older than 40 years in the Cambridge area who had a PSA test has increased from 1.4% in 1996 to 5.2% in 2002. The rate of diagnosis of prostate cancer was 45% higher in patients with prediagnosis PSA testing. More than half of the cases with prediagnosis PSA testing are likely to have been overdiagnosed, that is they would not have been detected clinically in their lifetime without the PSA testing.

The proportion of men 40 years and over in the Cambridge area having at least one prediagnosis PSA test in a given calendar year is comparable to the national figures. In the UK, a survey of the GP database showed that the proportion of men with no previous record of prostate cancer having at least one PSA test increased from 1.4% in 1994 to 3.5% in 1999 (Melia and Moss, 2001).

The relatively small percentage of prostate cancer cases, 5.6% (38 out of 680), between 1996 and 2002 with a recorded prediagnosis or peridiagnosis PSA test is surprising. However, the estimated number of excess cases (252) in East Anglia based on the results here is very close to the number estimated (289) based on age-period-cohort analysis reported in our previous study (Pashayan *et al*, 2006). There may be minor faults in record linkage between the cancer registry and the laboratory records for PSA testing and subsequent underestimation in the estimates of excess diagnosis associated with PSA testing. However, in this setting with a relatively small number of men actually receiving PSA tests, the estimates are at least plausible.

In the Cambridge area, prostate cancer was detected in only 0.4% (38 out of 8894) of men tested for PSA. Studies of screening for prostate cancer have shown widely varying detection proportions, from 0.2% (Perrin *et al*, 1991) to 4.8% (Smith and Catalona, 1994). Results vary according to the screening methods used (PSA, PSA with and without digital rectal examination, PSA with and without transrectal ultrasound), age of the participants, sample sizes, PSA cutoff points employed to determine further tests (Selley *et al*, 1997) and biopsy procedures and policies (Emiliozzi *et al*, 2004). Each of these factors can affect the detection proportion. Also, of course, the results in the literature pertain to population screening rather than increased diagnostic availability, as in this study. The much larger rates of detection in the screening studies are also partly due to the fact that rates have been reported for prevalence screen rather than for incidence screen. In the years following the prevalence screen, the average incidence rate will be smaller. In Ciatto *et al* (2005), prostate cancer was diagnosed in 1.75% of patients tested for PSA at the first screening and in 0.65% at the second.

In this study, a history of PSA testing was associated with a 45% excess risk of prostate cancer diagnosis. A randomised controlled study on screening effectiveness in Sweden showed that men undergoing active screening had a 2.6-fold increased risk of being diagnosed with prostate cancer during the 7-year study period (Hugosson *et al*, 2004). A study from Florence showed 66% increased risk of prostate cancer diagnosis in men having PSA screening over 9-year period (Ciatto *et al*, 2005). These results from screening tests suggest that the effect of prediagnosis PSA testing on incidence is more modest than that of formal population screening.

The unadjusted RR of 1.45 was used for the calculation of PSA-induced excess registration. This is not ideal. However, as there is age heterogeneity of the effect, the age-adjusted effect would be even less appropriate. The ideal would be to use age-specific RRs. However, the small number of cases in individual age groups would render these unstable. It is hoped that in future, larger studies with individual PSA data will be able to produce age-specific estimates.

The small numbers of PSA-exposed men in this study means that there is a considerable uncertainty in our estimates. We have not produced an age-specific or age-adjusted overdiagnosis estimate partly because of the small numbers and partly because of the interaction of the PSA effect with age, whereby the excess is observed at younger ages. A high excess at younger ages usually means that a standardised ratio will tend to be large. If we had sufficient data to estimate the age-specific excesses with precision, the overall excess might be larger than the unadjusted estimate.

Within the age structure of the study population, approximately 60% of the observed excess cases in the PSA-tested group are estimated to be overdiagnosed. These estimates pertain essentially to increased diagnostic use rather than population screening. In the latter case both excess incidence and overdiagnosis would be larger.

In this study, 12–31% of the cases, with a record of prediagnosis PSA, were estimated to be overdiagnosed cases. Etzioni *et al* (2002), using simulation, estimated overdiagnosis of 29–44% in 60–84 year-old men undertaking PSA screening. This estimation is dependent on the lead time and the remaining lifetime (which depends on the age of the individual). The lead time for an individual PSA detected case is a well-defined concept. It is the difference between the time the tumour is diagnosed as a result of PSA testing and the time it would have been diagnosed in the absence of PSA testing. It is difficult to estimate the lead time in the absence of an organised screening programme. In this study, an average of 7 years, which is typically reported in the literature, was assumed. It may be that because the situation in East Anglia is not one of formal screening, the earlier detection as a result of more widespread use of PSA gives a shorter lead time. Use of longer (10 years) and shorter (5 years) lead times made minor differences to the estimates.

Extrapolation of the Cambridge area findings to East Anglia showed that a 1.6% {(78/(252)^*^5.3%} excess of cases predicted for 1996–2000 could be attributed to PSA testing, which is around a quarter of the 6%, total excess of cases, estimated in our previous study. Approximately, 1% of all estimated cases in East Anglia would be classified as overdiagnosed. Thus, a majority of the recent excess is not explained by PSA testing.

In conclusion, this study shows that a substantial minority of the observed excess of cases in recent years is likely to be due to PSA testing. A majority of the observed excess in the PSA-tested group is estimated to be overdiagnosis. This, however, is a very small minority of the total number of cases diagnosed. Further quantification of the overdiagnosis will result from continued surveillance and from linkage of incidence to testing in other hospitals.

## Figures and Tables

**Table 1:** Number of prostate cancer registrations, men-years at risk, and incidence rate per 100 000 men-years, in men resident in the Cambridge area during 1996–2002, stratified by existence of a PSA test in this calendar period and before diagnosis

	**PSA tested**			**PSA not tested**		
**Age group (years)**	**No. of cases**	**Men-years at risk^a^**	**Incidence rate per 100 000 men-year**	**No. of cases**	**Men-years at risk^a^**	**Incidence rate per 100 000 men-year**
40–44	0	285	—	0	44784	—
45–49	0	474	—	1	43295	2
50–54	2	880	227	18	41339	44
55–59	3	1178	255	34	32623	104
60–64	3	1323	227	59	26565	222
65–69	7	1385	505	111	22614	491
70–74	7	1231	569	145	19924	728
75–79	5	1060	472	132	15585	847
80–84	3	679	442	98	9755	1005
85–89	2	401	499	50	5191	963
						
Total	32	8896	360^b^	648	261675	248^b^

PSA=prostate-specific antigen.

a Denominator estimates based on testing status at midyear.

b Crude incidence rate.

**Table 2:** Year of testing and age adjusted RR and age-specific RR and 95% CI for prostate cancer diagnosis in PSA tested *vs* nontested men resident in Cambridge area, 1996–2002

	**RR**	**95% CI**
*Adjusting variable*		
None	1.45	1.02–2.07
Year	1.39	0.98–1.99
Age group (two categories)	0.86	0.60–1.23
Year+age group	0.83	0.58–1.19
		
*Interaction variable*		
Age group (years)		
40–64	3.25	1.59–6.67
65–89	0.69	0.46–1.04

CI=confidence intervals; PSA=prostate-specific antigen RR=rate ratios.

**Table 3:** Estimated lead times for screen detected prostate cancer

**Paper**	**Age range**	**Lead time (years)**	**Study setting**
Tornblom *et al* (2004)	55–70	4.5	Population-based cohort study
			
Draisma *et al* (2003)	55	12.3	MISCAN model, based on estimates derived from the European randomised study of screening for prostate cancer
	60	11.0	
	65	9.5	
	70	7.7	
	75	6.0	
			
Etzioni *et al* (2002)	60–84	5.0	Modelling
			
Auvinen *et al* (2002)		7.0	Randomised trial (Finland)
			
Hugosson *et al* (2000)	67	7.0	Nested case–control study (Sweden)
			
McGregor *et al* (1998)	50–70	12.0	Modelling
			
Gann *et al* (1995)	40–84	5.5	Nested case–control study (USA)

**Table 4:** Expected remaining lifetime and probability of overdiagnosis based on lead times of 5, 7 and 10 years

**Age group (years)**	**Expected remaining lifetime (years)**	**Probability of overdiagnosis (%)**	**Probability of overdiagnosis (%)**	**Probability of overdiagnosis (%)**
		**M^a^=5 years**	**M^a^=7 years**	**M^a^=10 years**
40–44	35.8	0.08	0.6	2.8
45–49	31.2	0.2	1.2	4.4
50–54	26.7	0.5	2.2	6.9
55–59	22.4	1.1	4.1	10.6
60–64	18.4	2.5	7.2	15.9
65–69	14.7	5.3	12.2	23.0
70–74	11.4	10.2	19.6	32.0
75–79	8.6	17.9	29.3	42.3
80–84	6.3	28.4	40.7	53.3
85–89	4.5	40.7	52.6	63.8

Based on lifetable on UK males based on data for the years 2001–2003, produced by the Government Actuary's Department.

a M-mean sojourn time ∼ lead time.

**Table 5:** Estimated number of overdiagnosed cases using lead times of 5, 7 and 10 years, and number of excess cases for patients diagnosed in the Cambridge area from 1996–2002

**Age group (years)**	**No. of cases with prediagnosis PSA test**	**No. of excess cases**	**No. of overdiagnosed cases (if lead time=5 years)**	**No. of overdiagnosed cases (if lead time=7 years)**	**No. of overdiagnosed cases (If lead time=10 years)**
40–64	8	2.48	0.12	0.38	0.94
65–74	14	4.34	1.08	2.23	3.85
75–89	10	3.10	2.56	3.73	5.00
					
Total	32	9.92	3.74	6.34	9.77

PSA=prostate-specific antigen.
