# Excess cases of prostate cancer and estimated overdiagnosis associated with PSA testing in East Anglia

**DOI:** 10.1038/sj.bjc.6603330

**Published:** 2006-08-29

**Authors:** N Pashayan, J Powles, C Brown, S W Duffy

**Correction to:** *British Journal of Cancer* (2006) **95**, 401–405, doi:10.1038/sj.bjc.6603246

Owing to an author error, there was an erroneous statement presented in the Abstract of the above paper.

Where it was stated, ‘Assuming average lead times of 5 to 10 years, 40–64% of the PSA-detected cases were estimated to be overdiagnosed’, the figure 64% corresponds to average lead time of 7 years whereas, the corresponding figure for 10 years would be 98%.

The correct statement should read ‘Assuming average lead times of 5–10 years, 40–98% of the PSA-detected cases were estimated to be overdiagnosed’.

